# The effect of curcumax on postpartum women’s depression: a randomized controlled trial

**DOI:** 10.3389/fpsyt.2024.1302174

**Published:** 2024-09-10

**Authors:** Fatemeh Nikpour, Somayeh Ansari, Parvin Abedi, Shayesteh Jahanfar, Naeim Sharifat, Gholamreza Hooshmand, Elham Maraghi

**Affiliations:** ^1^ Midwifery Department, Reproductive Health Promotion Research Center, Ahvaz Jundishapur University of Medical Sciences, Ahvaz, Iran; ^2^ Midwifery Department, Menopause Andropause Research Center, Ahvaz Jundishapur University of Medical Sciences, Ahvaz, Iran; ^3^ Department of Public Health and Community Medicine, Tufts School of Medicine, Boston, MA, United States; ^4^ Psychiatry and Behavioral Center, Addiction Institute, Department of Pharmacology, Faculty of Medicine, Mazandaran University of Medical Sciences, Sari, Iran; ^5^ Psychiatry and Behavioral Sciences Research Center, Addiction Institute, Department of Pharmacology Faculty of Medicine, Mazandaran University of Medical Sciences, Sari, Iran; ^6^ Biostatistics Department, Public Health School, Ahvaz Jundishapur University of Medical Sciences, Ahvaz, Iran

**Keywords:** curcumax, postpartum depression, Edinburgh scale, reproductive-aged women, ginger, turmeric, black pepper

## Abstract

**Background:**

Postpartum depression is a major psychiatric disorder that affects the mother-baby attachment and may impair cognitive development of the child.

**Objective:**

This study aimed to evaluate the effect of curcumax (including ginger, turmeric, and black pepper) on postpartum depression in reproductive-aged women.

**Material and methods:**

This was a randomized controlled trial in which 124 women were recruited and randomly assigned into two groups of curcumax (n=62) and placebo (n=62) who consumed curcumax or placebo for 8 weeks (one capsule each day). Postpartum depression was measured using Edinburgh Depression Scale. Data were analyzed using Chi-square, independent t-test, and GEE.

**Results:**

The mean (SD) score of depression score was 15.83 (2.77) and 15.45 (2.97) before intervention, which reduced to 3.48 (4.29) and 7.22 (3.98) in the intervention and control groups, respectively after 4 weeks (p<0.0001). After eight weeks of intervention, these scores reduced to 1.72 (3.30) and 5.85 (3.67) in the intervention and control groups, respectively (p<0.0001).

**Conclusion:**

The results of this study showed that curcumax significantly reduced the mean score of postpartum depression among reproductive-aged women. Because it is the first time this herb was used as an anti-depressant, its effective dose was not available. Therefore, further studies with higher doses of this herb are recommended.

**Clinical Trial Registration:**

https://irct.behdasht.gov.ir/search/result?query=IRCT20210822052254N1, identifier IRCT20210822052254N1.

## 1 Introduction

Postpartum depression (PPD) is defined as a psychiatric disorder characterized by a drop in mood that can happen four weeks after birth until 30 weeks postpartum (1). Women who suffer from PPD may have symptoms such as depressed mood, irritability, episodes of crying without reason, feeling of tiredness, lack of concentration, and changes in sleep pattern (2). PPD can occur in women with different conditions such as high-risk pregnancy, a history of anxiety and premenstrual syndrome, dissatisfaction with the baby’s gender, and a history of sexual abuse (3). Insufficient social support (emotional and/or financial) may also cause PPD (4).

The prevalence of PPD has been reported differently in different parts of the world. A systematic review including 565 studies in different countries around the world showed that the prevalence of PPD is 17.22% with the highest rate being reported in South Africa (5). Another systematic review showed that the prevalence of PPD in Iran is 25.3%, which is almost doubled among women with a history of depression, illiterate women, and those having an undesired pregnancy (6). During the COVID-19 pandemic, the prevalence of PPD in Iran rose from 24.9% to 68.2% (7).

Untreated and severe PPD may delay the cognitive and language development of children and negatively affect the mother-baby attachment (8). A cohort study showed that women with PPD are more likely to show depression a few years after delivery and obtain low scores of general health in comparison to otherwise healthy women (9).

Diagnosis of PPD can be done using psychometric evaluation tests such as Edinburgh Postnatal Depression Scale (EPDS) (10), Postpartum Depression Screening Scale (PDSS), or Physician’s Health Questionnaire (11).

Treatment of PPD is often based on antidepressant medications such as fluoxetine (12). Other non-pharmacological methods such as cognitive behavioral therapy (13), electroconvulsive therapy (14), and bright light therapy are recommended for treatment of PPD (15).

Complementary and alternative medicine treatments include lavender (16), omega-3 fatty acids, folic acid, St. John’s Wort, exercise, and acupuncture (17). Curcumax is a mixture of three herbs namely turmeric, ginger, and black pepper. The ginger mitigates the pain by inhibiting lipoxygenase and cyclooxygenase and decreasing the secretion of prostaglandin (18). Curcumin derived from rhizome of *Curcuma longa* has many properties such anti-inflammatory, antioxidant, and anti-depressant effects (19). Black pepper, derived from *Piper nigrum L*, is a food spice that has been reported to have antidiabetic, hepatoprotective and neuroprotective effects (20). Although there is evidence suggesting the neuroprotective effect of ginger, turmeric and black pepper, there is little, if any, evidence showing the effect of their combination. Therefore, this randomized controlled trial was designed to evaluate the effect of curcumax on postpartum depression of reproductive-aged Iranian women.

## 2 Methods

This was a randomized controlled trial on 124 women with postpartum depression. The design of the study was approved by the Ethics Committee of Ahvaz Jundishapur University of Medical Sciences (Ref. No: IR.AJUMS.REC.1400.336). The protocol of the study was registered in the Iranian Registry of Randomized Controlled Trials (Code No: IRCT20210822052254N1). All participants provided written informed consent prior to data collection.

### 2.1 Sample size

The sample size for this study was determined using the below formula based on comparing the means of two independent groups. In a previous study (21), the standard deviation for the EPDS's score (s) at the end of the study was reported as 7.0. The mean difference between the two groups (d) was considered to be 4.0. Using these values, with an alpha level of 0.05, a power of 0.85, and accounting for a 10% attrition rate, the required sample size for each group was calculated to be at least 62 pregnant women.


$$\text{n}=\frac{{\left(Z_{1-\frac{\alpha }{2}}+Z_{1-\beta }\right)}^{2}\left(2s^{2}\right)}{{\left(\text{d}\right)}^{2}}=\frac{\left(1.96^{2}+1.04^{2}\right)\times 2\times 7^{2}}{{\left(4\right)}^{2}}≅55$$

### 2.2 Inclusion/exclusion criteria

Women aged 18-35 who obtained scores between 12-23 from Edinburgh Postnatal Depression Scale (EPDS), had basic literacy, had a term and low risk pregnancy with a healthy neonate, and whose childbirth was 6 months prior to data collection were recruited for this study. Women with the following conditions were excluded from the study: allergy to herbal medicine, substance abuse, or severe depression. Women with severe depression and suicidal thoughts were referred to a psychiatrist.

### 2.3 Randomization and allocation concealment

For randomization, we used permuted block randomization technique with a block size of 6 and an allocation ratio of 1:1. The code dedicated to each group was written on a piece of paper and kept with the ward clerk of health center. Therefore, neither the researcher nor the participants knew about group allocation. Both the researcher who distributed medicines and the participants were blinded.

### 2.4 Setting

A health clinic (No 3) located in the west bank of Karun River in Ahvaz city was used for data collection. Ahvaz is the capital of Khuzestan province and has a population of 1,136,989 people.

### 2.5 Instruments

A demographic questionnaire and Edinburgh Postnatal Depression Scale (EPDS) were used to collect the data. The demographic questionnaire included questions about age, education, occupation, economic status, length of marriage, mode of delivery, and husband’s age, education, and occupation. The content validity of the demographic questionnaire was evaluated and confirmed.

EPDS has 10 questions scored based on a four-point scale: from 0: “never” to 3 for “often”. The minimum total score is zero and maximum total score is 30 (22). This scale can be used for the detection of postnatal depression from six weeks after delivery onward. The Persian version of this questionnaire was validated by KaniGolzar et al. in Iran (23).

### 2.6 Intervention

The Curcumax and placebo capsules were made in Pharmacology school of Ahvaz Jundishapur University of Medical Sciences. The curcumax capsules contain turmeric (the dried root powder, 320 mg), ginger (150 mg), and black pepper (4mg). Each placebo capsules (600 mg) contained Avicel (300 mg), Starch (258 mg), Gelatin (39 mg), Magnesium Stearate (3mg).

An equal number of curcumax and placebo capsules to be used for 8 weeks were prepared in separate containers that were identical in terms of shape, color, and size. The containers were coded by a person who was not aware of nature of the study. After randomization, each drug container was given to a participant, and the corresponding code was recorded. Participants were advised to take one curcumax or placebo capsule each day for 8 weeks. In case of they had any question or experienced any the side effect, they could call the researcher.

### 2.7 Follow-up

Four and eight weeks after initiation of intervention, the EPDS questionnaire was completed by the participants in both groups.

### 2.8 Statistical analyses

All data were imported into SPSS version 22. Quantitative variables were reported as mean and standard deviation, minimum and maximum. The qualitative variables were reported as numbers and percentages. The normality of quantitative variables was checked using the Shapiro-Wilk test. Chi-square test or Fisher’s exact test was used to examine the relationship between qualitative variables, and the Mann-Whitney test was used to compare quantitative variables between the two independent groups.

Association between changes in the EPDS's score over time and treatment groups (intervention, placebo) was examined by using generalized estimating equation (GEE) model. The GEE model consists of main effects (for treatment group, time, adjusted variables) and the interaction effect of time and treatment group. Three time points were considered in the analyses as baseline, week 4 and week 8. Pairwise comparisons after Bonferroni correction followed by GEE analysis. All tests were two sided and P-values < 0.05 were considered statistically significant.

## 3 Results

**Figure 1:**
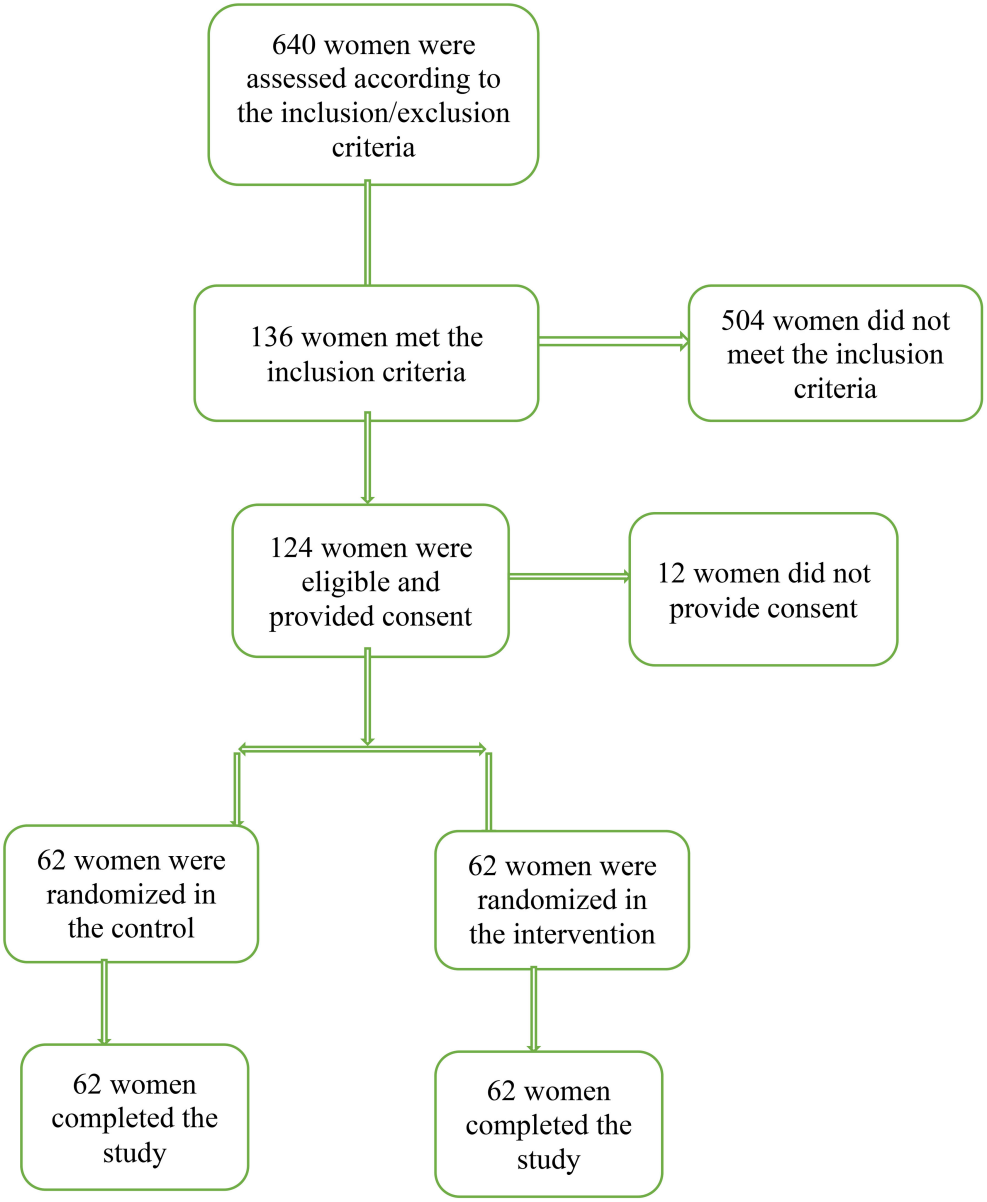
Recruitment and retention of participants in the study.

In this study, 124 women were recruited and randomized into two groups of curcumax and control. All women in both groups completed eight weeks of follow-up (**Figure 1**). The mean age of women in the curcumax and control groups was 26.12 ± 4.44 and 23.95 ± 4.65 years respectively. Most women 24(38.7%) in the intervention group had a diploma, while most women in the control 23(37.1%) group had high school education. Two groups showed a significant difference (p=0.001). The same was true with husbands. That is, 25(40.3%) husbands in the intervention group had a diploma, while most 21(33.9%) husbands in the control group had high school education. Two groups showed a significant difference regarding education of their husbands (p=0.014). Most women in the two groups were house-wives and had a moderate economic status (**Table 1**).

**Table 1:** Demographic characteristics of women in curcumax and control groups.

Variables	Intervention N=62	Control N=62	Statistics	P-value
	Mean ± SD		Z	
Age (y)	26.12 ± 4.44	23.95 ± 4.65	-2.73	0.006
Age of husband (y)	30.33 ± 5.88	28.66 ± 4.48	-1.64	0.100
Age of last child (m)	3.24 ± 1.37	3.16 ± 1.36	-0.44	0.659
Marriage age (y)	1.51 ± 0.59	1.40 ± 0.95	-1.89	0.058
	N (%)		Chi-Square	
**Education**			19.67	0.001
Primary	6 (9.7)	9 (14.5)		
High school	13 (21)	23 (37.1)		
Secondary high school	8 (12.9)	16 (25.8)		
Diploma	24 (38.7)	14 (22.6)		
University education	11 (17.7)	0 (0)		
**Education of husband**			12.47	0.014
Primary	8 (12.9)	4 (6.5)		
High school	13 (21)	21 (33.9)		
Secondary high school	7 (11.3)	18 (29)		
Diploma	25 (40.3)	15 (24.2)		
University education	9 (14.5)	4 (6.5)		
**Occupation**			0.0	0.976
Housewife	60 (96.8)	60 (96.8)		
Employee	2 (3.2)	2 (3.2)		
**Economic status**			0.72	0.585
Weak	18 (29)	14 (22.6)		
Moderate	33 (53.2)	37 (59.7)		
Good	11 (17.7)	11 (17.7)		
**Number of children**			0.13	0.856
1	26 (41.9)	28 (45.2)		
2	36 (58.1)	34 (54.8)		

**Table 2** shows the mean (SD) of postpartum depression in the intervention and control groups. As evident in this table, the mean score of depression reduced significantly more in the intervention group compared to the control group after 4 weeks (the mean (SD) score of depression was 15.83 (2.77) in the intervention group that reduced to 3.48 (4.29) and 1.72 (3.30) after 4- and 8- weeks intervention respectively. The mean (SD) score of depression was 15.45 (2.97) at baseline in the control group that reduced to 7.22 (3.98) and 5.85 (3.67) after 4- and 8-weeks intervention respectively. These differences were significant in two groups over the time using the generalized estimating equations (GEE) (p<0.0001, **Table 3**). The Bonferroni-corrected pairwise comparison following generalized estimating equation (GEE) showed that difference between control and curcumax was significant in week 4 (mean=3.97, 95% CI: 2.09, 5.84, p<0.0001) and week 8 (mean=4.35, 95% CI: 2.77, 5.94, P<0.0001) (**Table 4**). None of the participants experienced any side effects.

**Table 2:** Summary of study outcomes at baseline, week 4 and week 8 in the two study groups.

Outcome	Baseline	Week 4	Week 8
EPDS score			
Control group			
* Mean (SD, range)*	15.45 (2.97, 11.0-22.0)	7.22 (3.98, 0.0-18.0)	5.85 (3.67, 0.0-20.0)
* Median (IQR)*	15.0 (13.0-17.0)	8.0 (5.0-10.0)	6.0 (3.75-8.0)
Curcumax group			
* Mean (SD, range)*	15.83 (2.77, 11.0-23.0)	3.48 (4.29, 0.0-20.0)	1.72 (3.30, 0.0-18.0)
* Median (IQR)*	15.0 (14.0-18.0)	2.0 (0.0-5.25)	0.0 (0.0-3.0)

EPDS, Edinburg Postpartum Depression Scale; IQR, interquartile range (25^th^–75^th^ percentiles).

**Table 3:** Estimated outcomes over time between the two groups according to the generalized estimating equations (GEE) test.

Outcome, mean (*SE*)	Time points			Statistics	Statistics	Statistics
	Baseline	Week 4	Week 8	P-value_Time_	P-value_Group_	P-value_Time×Group_
EPDS's score				1118.07	38.49	39.92
* Curcumax group*	15.74 (0.38)	3.38 (0.51)	1.63 (0.38)	< 0.0001	< 0.0001	< 0.0001
CI 95 % for mean	(14.98 to 16.50)	(2.38 to 4.39)	(0.87 to 2.38)			
* Control group*	15.58 (0.50)	7.36 (0.56)	5.99 (0.53)			
CI 95 % for mean	(14.60 to 16.56))	(6.25 to 8.47))	(4.94 to 7.03)			

EPDS, Edinburg Postpartum Depression Scale; GEE, generalized estimating equations; CI, Confidence Interval; Adjusting for the women’s age, women and their husband’s education, and length of marriage as fixed factors was considered. Wald statistics is reported for main effects and the interaction effect between time and treatment group.

**Table 4:** Bonferroni-corrected pairwise comparisons following generalized estimating equations (GEE) analysis.

Outcome	Control group		Curcumax group		Time point	Difference (control minus Curcumax)	
	Mean (95% CI)	P-value	Mean (95% CI)	P-value		Mean (95% CI)	P-value
EPDS's score							
Week 0 minus Week 4	8.22 (6.48 to 9.96)	< 0.0001	12.35 (10.70 to 14.00)	< 0.0001	Week 0	-0.15 (-1.85 to 1.54)	> 0.99
Week 0 minus Week 8	9.59 (7.97 to 11.21)	< 0.0001	14.11 (12.77 to 15.45)	< 0.0001	Week 4	3.97 (2.09 to 5.84)	< 0.0001
Week 4 minus Week 8	1.37 (0.46 to 2.27)	< 0.0001	1.75 (.82 to 2.69)	< 0.0001	Week 8	4.35 (2.77 to 5.94)	< 0.0001

## 4 Discussion

This study was designed to evaluate the effect of curcumax on postpartum depression in reproductive-aged women. The results showed that the mean score of depression was decreased significantly in the intervention group in comparison to the control group after 4 and 8 weeks follow-up. We could not find any study evaluating the effect of curcumax on postpartum depression. Therefore, we mention studies that evaluated the effect of each ingredient of curcumax. One of the mechanisms of reducing postpartum depression by ginger and turmeric is through their anti-inflammatory effects, and there is some evidence that dietary inflammation index has a relationship with postpartum depression (24).

It is also well documented that ginger could suppress 5-lipooxygenase or prostaglandin, and this suppression can not only inhibit prostaglandin and leukotriene synthesis bur also reduce the level of pro-inflammatory cytokines such as IL-1, TNF-α and IL-8 (25). In a systematic review on 14 studies, Askari et al. found that ginger supplementation could reduce C-Reactive protein, IL-6, and Tumor Necrosis Factor (TNF- α), and increase blood total antioxidant capacity (TAC) significantly (26).

On the other hand, in their systematic review including 10 studies, Matias et al. found that curcumin (component of turmeric) could significantly increase monoamines and brain-derived neurotrophic factor and suppress the production of pro-inflammatory cytokines and neural apoptosis in brain, thus mitigating depression and anxiety (27). In a randomized controlled trial including 65 patients with major depression who were randomized into two groups of curcumin as an adjunctive therapy and placebo, Kanchanatawan et al. found that curcumin could improve the symptoms of major depression in weeks 12 and 16 compared to placebo (28). The anti-inflammatory and anti-arthritic effect of piperine (the active component of black pepper) on rats was evaluated by Bang et al. Their results showed that piperine could suppress the expression of IL6 and matrix metalloproteinase (MMPs), and therefore reduce the synthesis of prostaglandin E2, the symptoms of arthritis, and inflammatory areas in the ankle joint (29). Furthermore, Nagaraju et al. in a study on rats evaluated the anti-inflammatory effect of turmeric, alma (Emblica Officinalis Gaertn), and black pepper (TAB) on sepsis-induced acute lung injury. Their results showed that TAB could significantly decrease the inflammatory cytokines including tissue necrosis factor (TNF) and IL6 in the blood, and improve histopathological changes in the lungs (30). As the curcumin is a component of turmeric which represents about 2-8% of most preparations, the results of Nagaraju et al’s study can support the results of the present study.

A mentioned earlier, we could not find any articles directly dealing with the effect of curcumax on postpartum depression. However, all these mechanisms related to the three ingredients of curcumax capsules show that this substance has anti-inflammatory properties and can change the depression pathways.

### 4.1 Strengths and limitations of the study

This is the first study to evaluate the combined effect of ginger, turmeric, and black pepper in one capsule on reducing postpartum depression. Despite its merits, this study has some limitations. In this study, we did not measure the blood level of depression markers. Measuring such markers could show a more precise picture of drug effect. We conducted this study at the time of COVID-19 pandemic, which may have increased the level of anxiety, stress, and depression among the studied women. And finally, the curcumax capsules that used in this study contained turmeric (320 mg), ginger (150 mg), and black pepper (4mg), and we followed participants until eight weeks. Further research with different doses of curcumax and longer duration are recommended.

## 5 Conclusion

The results of this study showed that curcumax significantly reduced the mean score of postpartum depression among reproductive-aged women. Because it is the first time this herb is used as an anti-depressant, its effective dose was not available. Therefore, further studies with higher doses of this herb are recommended.

## Data Availability

The raw data supporting the conclusions of this article will be made available by the authors, without undue reservation.

## References

[1] Andrews-Fike C. A review of postpartum depression. Prim Care Companion J Clin Psychiatry. (1999) 1:9–14. doi: 10.4088/pcc.v01n0103 15014700 PMC181045

[2] Rich M. What is postpartum depression. UNICEF. Available online at: https://www.unicef.org/parenting/mental-health/what-postpartum-depression#postpartum-depression (Accessed 16 September 2023).

[3] Mughal S Azhar Y Siddiqui W. Postpartum depression. Available online at: https://www.ncbi.nlm.nih.gov/books/NBK519070/ (Accessed 7 October 2022).

[4] Escribà-Agüir V Artazcoz L. Gender differences in postpartum depression: A longitudinal cohort study. J Epidemiol Community Health. (2011) 65:320–6. doi: 10.1136/jech.2008.085894 PMC3069755 20515899

[5] Wang Z Liu J Shuai H Cai Z Fu X Liu Y. Mapping global prevalence of depression among postpartum women. Transl Psychiatry. (2021) 11:543. doi: 10.1038/s41398-021-01663-6 34671011 PMC8528847

[6] Veisani Y Delpisheh A Sayehmiri K Rezaeian S. Trends of postpartum depression in Iran: a systematic review and meta-analysis. Depress Res Treat. (2013) 2013:291029. doi: 10.1155/2013/291029 23936640 PMC3722792

[7] Afshari P Tadayon M Abedi P Beheshtinasab M. Comparison of pre-and intra-COVID-19 postpartum depression among reproductive aged women: A comparative cross-sectional study in Ahvaz Iran. Front Psychiatry Sec. Perinatal Psychiatry. (2022) 13:1019432. doi: 10.3389/fpsyt.2022.1019432 PMC9679291 36424994

[8] Netsi E Pearson RM Murray L Cooper P Craske MG Stein A. Association of persistent and severe postnatal depression with child outcomes. JAMA Psychiatry. (2018) 75:247–53. doi: 10.1001/jamapsychiatry.2017.4363 PMC5885957 29387878

[9] Abdollahi F Zarghami M. Effect of postpartum depression on women’s mental and physical health four years after childbirth. EMHJ. (2018) 24:1–13. doi: 10.26719/2018.24.10.1002 30582143

[10] Cox J Holden J Sagovsky R. Detection of postnatal depression: development of the 10-item Edinburgh Postnatal Depression Scale. Br J Psychiatry. (1987) 150:782–6. doi: 10.1192/bjp.150.6.782 3651732

[11] Hanusa BH Scholle SH Haskett RF Spadaro K Wisner KL. Screening for depression in the postpartum period: a comparison of three instruments. J Womens Health (Larchmt). (2008) 17:585–96. doi: 10.1089/jwh.2006.0248 PMC7083208 18345995

[12] Appleby L Warner R Whitton A Faragher B. A controlled study of fluoxetine and cognitive-behavioural counseling in the treatment of postnatal depression. BMJ. (1997) 314:932–6. doi: 10.1136/bmj.314.7085.932 PMC2126383 9099116

[13] Cooper PJ Murray L Wilson A Romanuk H. Controlled trial of the short- and long-term effect of psychological treatment of postpartum depression. Br J Psychiatry. (2003) 182:412–9. doi: 10.1192/bjp.182.5.412 12724244

[14] Forray A Ostroff RB. The use of electroconvulsive therapy in postpartum affective disorders. J ECT. (2007) 23:188–93. doi: 10.1097/yct.0b013e318074e4b1 17804998

[15] Epperson CN Terman M Terman JS. Randomized clinical trial of bright light therapy for antepartum depression: preliminary findings. J Clin Psychiatry. (2004) 65:3. doi: 10.4088/JCP.v65n0319 15096083

[16] Babakhanian M Rashidi Fakari F Mortezaee M Bagheri Khaboushan E Rahimi R Khalili Z. The effect of herbal medicines on postpartum depression, and maternal-infant attachment in postpartum mother: A systematic review and meta-analysis. Int J Pediatr. (2019) 7:9645–56. doi: 10.22038/ijp.2019.38414.3298

[17] Deligiannidis KM Freeman MP. Complementary and alternative medicine therapies for perinatal depression. Best Pract Res Clin Obstet Gynaecol. (2014) 28:85–95. doi: 10.1016/j.bpobgyn.2013.08.007 24041861 PMC3992885

[18] Pourmaleky S Najar S Montazery S Haghighizadeh MH. Comparison between the effect of zintoma (Ginger) and mefenamic acid on after pain during postpartum in multioarous women. Iran J Obstet Gynecol Infertil. (2013) 16:18–25.

[19] Matrisciano F Epinna G. PPAR and functional foods: rationale for natural neurosteroid-based interventions for postpartum depression. Neurobiol Stress. (2020) 12:100222. doi: 10.1016/j.ynstr.2020.100222 32426424 PMC7226878

[20] Balakrishnan R Azam S Kim IS Choi DK. Neuroprotective effects of black pepper and its bioactive compounds in age-related neurological disorders. Aging Dis. (2023) 14:750–77. doi: 10.14336/AD.2022.1022 PMC10187688 37191428

[21] Mahdizadeh A Tafazoli M Mazloum SR Manteghi A Asili J Noras MR. Effect of orange scent on preventing of postpartum depression: A randomized clinical trial. Iran. J Obstet. Gynecol. Infertil. (2018) 21:93–100. doi: 10.22038/ijogi.2018.12139

[22] Schaper A Rooney B Kay N Silva P. Use of the Edinburgh Postnatal Depression Scale to identify postpartum depression in a clinical setting. J Reprod Med. (1994) 39:620–4. 7996526

[23] Ahmadi kani Golzar A GoliZadeh Z. Validation of Edinburgh Postpartum Depression Scale (EPDS) for screening postpartum depression in Iran. J Nurs Edu. (2015) 3:1–10.

[24] Zou H Sun M Liu Y Xi Y Xiang C Yong C. Relationship between dietary inflammatory index and postpartum depression in exclusively breastfeeding women. Nutrients. (2022) 14:5006. doi: 10.3390/nu14235006 36501036 PMC9738724

[25] Tjendraputra E Tran VH Liu-Brennan D Roufogalis BD Duke CC. Effect of ginger constituents and synthetic analogues on cyclooxygenase-2 enzyme in intact cells. Bioorganic Chem. (2001) 29:156–63. doi: 10.1006/bioo.2001.1208 11437391

[26] Askari G Aghajani M Salehi M Najafgholizadeh A Keshavarzpour Z Fadaei A. The effects of ginger supplementation on biomarkers of inflammation and oxidative stress in adults: A systematic review and meta-analysis of randomized controlled trials. J Herb Med. (2020) 22:100364. doi: 10.1016/j.hermed.2020.100364

[27] Matias JN Achete Gm Campanari GS dos S Araujo AC Buglio DS. A systematic review of the antidepressant effects of curcumin: Beyond monoamines theory. Aust N Z J Psychiatry. (2021) 55:451–62. doi: 10.1177/0004867421998795 33673739

[28] Kanchanatawan B Tangwongchai S Sughondhabhiron A Suppapitipom S Hemrunrojn S Carvalho AF. Add-on treatment with curcumin has antidepressive effects in Thai patients with major depression: results of a randomized double-blind placebo-controlled study. Neurotox Res. (2018) 33:621–33. doi: 10.1007/s12640-017-9860-4 29327213

[29] Bang JS Oh DH Choi HM Sur BJ Lim SJ Kim JY. Anti-inflammatory and antiarthritic effects of piperine in human interleukin 1beta-stimulated fibroblast-like synoviocytes and in rat arthritis models. Arthritis Res Ther. (2009) 11:R49. doi: 10.1186/ar2662 19327174 PMC2688199

[30] Nagaraju M Kalahasti KK Reddy KP. Anti-inflammatory potential of turmeric, amla, and black pepper mixture against sepsis-induced acute lung injury in rats. J Food Sci Technol. (2023) 60:252–61. doi: 10.1007/s13197-022-05610-1 PMC9633023 36349282

